# Hypertension‐induced remodelling: on the interactions of cardiac risk factors

**DOI:** 10.1113/JP273043

**Published:** 2017-03-30

**Authors:** Jakub Tomek, Gil Bub

**Affiliations:** ^1^ Department of Physiology Anatomy and Genetics University of Oxford Oxford UK; ^2^ Department of Physiology McGill University Canada

**Keywords:** arrhythmia, extracellular matrix, heart failure, hypertension

## Abstract

Hypertension induces considerable cardiac remodelling, such as hypertrophy, interstitial fibrosis, and abnormal activity of the cardiac sympathetic nervous system, which are established risk factors in several highly dangerous heart diseases, such as ventricular fibrillation and congestive heart failure. All these risk factors and heart diseases are studied extensively in isolation, but to our knowledge, there is no comprehensive review of their interactions. At the same time, there is growing evidence suggesting that such interactions are numerous and that a successful therapy against a particular condition may have unexpectedly weak effects on mortality, as treated patients may die of a different cause exacerbated by the therapy. In this article, we present a multiscale review of the literature focusing on the relationships between the above‐mentioned risk factors and heart diseases, and introduce a framework that gives insight into their possible interactions. We use this framework to demonstrate that conditions such as fibrosis and elevated activity of the sympathetic nervous system may be compensatory, rather than purely pathological, mechanisms in certain contexts. Finally, we show why the described mechanisms are relevant not only in hypertension, but also in the case of healed myocardial infarction.

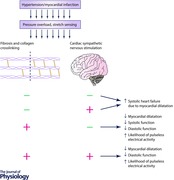

**Abbreviations**
Ang‐II: angiotensin‐II
CSNS: cardiac sympathetic nervous stimulation
ECM: extracellular matrix
MMP: matrix metalloproteinase
PEA: pulseless electrical activity
SCD: sudden cardiac death
TGF‐β: transforming growth factor β
VF: ventricular fibrillation

## Introduction

Hypertension is a major clinical condition that promotes extensive cardiac remodelling, acting as a contributing factor in both systolic and diastolic dysfunction, arrhythmias and symptomatic heart failure, which are among the main sources of mortality worldwide (Drazner, 2011). Through an analysis of the literature, we highlight the connections between hypertension‐induced remodelling and heart diseases, with a focus on the links between arrhythmogenesis, systolic and diastolic heart failure. Systolic heart failure, also known as heart failure with reduced ejection fraction, is characterized by poor contractile function and is usually associated with myocardial dilatation. Diastolic heart failure, also known as heart failure with preserved ejection fraction, is characterized by poor relaxation of ventricles during diastole (e.g. due to high myocardial stiffness) resulting in poor refilling. We discuss how both types of heart failure may be promoted by hypertension, but via different mechanisms.

We first focus on how hypertension induces structural cardiac remodelling and how this remodelling may be cardioprotective in certain contexts. In the second section, we review studies that suggest that hypertrophy is not a sufficient reaction against hypertension and that interstitial fibrosis and collagen crosslinking may play a key role in protecting the heart from systolic heart failure, even though they promote diastolic dysfunction and thus increase the likelihood of diastolic heart failure. In the third section, we review how cardiac structural remodelling and subsequent myocardial stiffness are linked to cardiac sympathetic nervous stimulation (CSNS) and how this is linked to ventricular fibrillation, pulseless electrical activity, and diastolic heart failure. In the fourth section, we show how the observations emerging from this review are relevant not only to hypertension, but also to understanding myocardial infarction. Ultimately, in the fifth section, we summarize the observations made and discuss their implications.

## From hypertension to fibrosis

Left ventricular hypertrophy, defined as an abnormal increase in left ventricular mass, is traditionally considered to be an adaptation to increased cardiac workload in hypertension via minimization of wall stress and normalization of ejection performance (Drazner, 2011). However, it is also associated with adverse effects, which is why it is often labelled as maladaptative (Frey & Olson, 2003).

Less attention seems to be paid to diffuse myocardial fibrosis, characterized by an increased deposition of extracellular matrix (ECM), possibly due to the difficulty of isolating fibrosis from hypertrophy, as they tend to occur together in hypertension. While the compensatory role of hypertrophy is acknowledged in the literature (Drazner, 2011), diffuse fibrosis is almost invariably considered to be pathological, mainly due to increased likelihood of fibrillation and increased ventricular stiffness leading to systolic or diastolic dysfunction (Creemers & Pinto, 2011). Below, we summarize studies pointing towards a cardioprotective role of diffuse fibrosis in protecting the heart from dilatation in a pressure overload setting, possibly because the stiff collagen scaffolding associated with fibrosis may mechanically prevent elongation of myocytes during myocardial dilatation. We also discuss how myocardial stiffening is related to systolic and diastolic heart failure.

### Systems‐level evidence

In an experimental study that used aortic banding to induce pressure overload in rats, Woodiwiss *et al*. (2001) divided treated animals into two groups, those with non‐failing hearts and those with hearts failing due to dilatation and systolic dysfunction. The authors showed a marked decrease in collagen crosslinking in the group with failing hearts, while total collagen content and the degree of hypertrophy were similar between the two groups. The first important observation in this study is that hypertrophy itself does not seem to be sufficient protection against persistent hypertension. A second observation is that the component of fibrotic remodelling that might offer protection against dilatation is collagen crosslinking, which has been shown to be the main factor in determining myocardial stiffness (Badenhorst *et al*. 2003), rather than total collagen content. Therefore, if there is to be a protective effect of fibrosis, the collagen produced needs to be appropriately crosslinked to prevent dilatation.

The work by Janicki and Spinale is another demonstration of the importance of fibrosis, showing that in the Syrian hamster, matrix metalloproteinase (MMP) activity and subsequent decrease of collagen volume fraction paralleled significant ventricular dilatation and heart failure, presumably due to systolic dysfunction (Janicki *et al*. 2006). MMP activity and its effect (breaking collagen and collagen crosslinks) has also been demonstrated in human studies of heart failure (Gunja‐Smith *et al*. 1996) and these studies therefore provide support for the view that stiff collagen scaffolding is a barrier to dilatation.

Kim *et al*. (2000) produced a genetically modified mouse model that manifests a chronic overexpression of MMP‐1. The modified animals, without an artificial increase in blood pressure, demonstrated hypertrophy and increased collagen content with subsequent reduction of collagen content, dilatation, and loss of systolic and diastolic function.

Using a different genetic modification, Takeda *et al*. (2010) investigated the effect of fibroblast‐specific deletion of the *Klf5* gene in murine hearts subject to previous transverse aortic constriction, demonstrating that fibroblasts (and *Klf5*) play a key role in response to pressure overload. While fibroblast‐specific deletion of *Klf5* ameliorated fibrosis and hypertrophy following pressure overload, it also led to left ventricular dilatation and early death.

An alternative style of inducing ventricular dilatation and subsequent heart failure is rapid pacing, as shown by McElmurray *et al*. (1999) in pigs. In their study, the authors show that inhibition of MMP increases myocardial stiffness, ameliorates the degree of ventricular dilatation and leads to a decrease in fractional shortening. This study is important as it shows that a possible benefit of increased cardiac stiffness may not be bound to dilatation necessarily due to hypertension, but also due to other mechanisms.

Another study of MMP inhibitors is the one by Peterson *et al*. (2001), in a rat model of heart failure, where the inhibition of MMP prevented development of heart failure due to dilatation. This study suggests the importance of the time scale of collagen remodelling during heart failure, since the collagen content increases in failing hearts. This observation raises the question: if fibrosis offers protection against dilatation, why is collagen abundant in dilated cardiomyopathy? The answer might be, as mentioned above, that the collagen crosslinking is the key factor, provided that there is enough collagen to be crosslinked. Indeed Gunja‐Smith *et al*. (1996) showed that crosslinking is decreased in dilated cardiomyopathy, most likely due to the activity of MMPs. Therefore, we might hypothesize that the overproduction of collagen in failing hearts is a dysfunctional remnant of an originally cardioprotective mechanism (increasing collagen content and collagen crosslinking to increase ventricular stiffness), where, for a reason not yet understood, the formation of crosslinks is now diminished, even while collagen continues to be produced.

Interpreting the above‐summarized studies together, we can see that the two variants of heart failure (with reduced or preserved systolic function) may be viewed as two sides of the same coin: the ventricular stiffness. That is, if the heart does not stiffen in the presence of pressure overload, it is likely to dilate due to increased stretch; however, if it does stiffen it will nevertheless diminish the capability of the ventricle to relax, which is why the heart may develop diastolic dysfunction and eventually diastolic heart failure.

### Cellular‐level evidence

It is well known that the collagen network in heart is not static, but undergoes continuous synthesis and degradation (Bishop & Laurent, 1995). If we focus on studies suggesting a protective effect of fibrosis in hypertension, we might ask if there are any mechanisms on a cellular and subcellular level that would promote such remodelling. Indeed, such mechanisms exist and are extensively researched; advances in this field are summarized in McCain & Parker (2011), which focuses on stretch sensing in myocytes, and Fan *et al*. (2012), which focuses on stretch sensing in fibroblasts and pro‐fibrotic signalling. In the context of this review, the key points are the following: (1) stretch promotes the release of growth factors such as TGF‐β and angiotensin II (Ruwhof & Laarse, 2000), both of which are pro‐fibrotic (González *et al*. 2002; Leask & Abraham, 2004); and (2) at the same time, TGF‐β1 upregulates lysyl oxidase, which promotes formation of collagen crosslinks (McCormick & Thomas, 1998). Therefore, there are known mechanisms both for stretch‐upregulated production of collagen and its crosslinking.

The signalling pathways involved in fibrotic remodelling are far from simple and may be cell‐type dependent. In the work of Engebretsen & Tønnessen (2014) in mice, broad β‐inhibition ameliorated fibrosis in pressure overload, but resulted in increased cardiac dilatation and ultimately mortality. Similar results were obtained using a different method of broad β‐signalling inhibition (Koitabashi *et al*. 2011).

**Figure 1:**
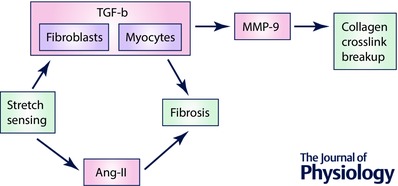
An overview of key pro‐fibrotic signals and the crosslink‐breakup signalling of TGF‐β in myocytes Ang‐II, angiotensin‐II; MMP‐9, matrix metalloproteinase 9; TGF‐β, transforming growth factor β.

On the other hand, in the study by Koitabashi *et al*., an inhibition of TGF‐β2 specifically in myocytes did not introduce dilatation, while improving cardiac function. Rainer *et al*. (2014) have similarly shown that after myocardial infarction, broad TGF‐β‐blockade has increased mortality due to wall rupture after dilatation, while myocyte‐specific TGF‐β‐blockade was beneficial for survival in the short term, decreasing the likelihood of wall rupture. Intriguingly, the existing literature suggests that myocyte‐specific TGF‐β2‐blockade is not only anti‐fibrotic, but also promotes ventricular stiffening: one of the effects of myocyte‐specific TGF‐β‐blockade was observed to be the attenuation of MMP‐9, activation of which is considered to be an important factor in transition from compensated hypertrophy to heart failure due to dilatation (Yabluchanskiy *et al*. 2013). At the same time, such a transition is mainly characterized by loss of collagen crosslinking, rather than of total collagen content (Woodiwiss *et al*. 2001). It is also known that MMP‐9 activation plays a role in formation of abdominal aortic aneurysms, a condition caused by insufficient strength of aortic wall (Wilson *et al*. 2008). We could therefore speculate that MMP‐9 activation decreases tissue stiffness and that its inhibition by myocyte‐specific beta‐blockade allows formation of stronger (presumably more crosslinked) collagen that prevents aortic or myocardial rupture, which provides a mechanism of beneficial effects seen in the studies by Rainer *et al*. and Koitabashi *et al*. In addition, a recent study in post‐infarction MMP‐9‐null mice demonstrated attenuated left ventricular dilatation, increased tissue stiffening, and a considerable increase in lysyl oxidase (and thus collagen crosslinking), even though collagen deposition was decreased (Voorhees *et al*. 2015). A visual summary of the observations above is given in Fig. 1.

The apparently opposite reaction to broad *versus* myocyte‐specific beta‐blockade suggests that there might be a competition between stretch‐sensing mechanisms in myocytes (promoting crosslink breakup via MMP‐9 activation) and fibroblasts (promoting cardiac stiffening and fibrosis). We might speculate that this competition could underlie how the myocytes sensing functional overload may decrease the myocardial integrity in order to elongate in an originally compensatory role according to the Frank–Starling mechanism; it is also possible that hypertrophy of the heart requires collagen crosslink breakup to accommodate angiogenesis (Yabluchanskiy *et al*. 2013). Then, when the myocyte performance matches the functional overload, the myocyte‐specific inhibition of structural integrity may stop and the other arm of this system, fibroblasts, may promote stiffening in order to maintain ECM integrity. At the same time, when the functional overload is severe and the Frank–Starling mechanism combined with hypertrophy does not compensate for the increased load, the collagen breakup signalling from myocytes may lead to critical destabilization of ECM, leading to ventricular dilatation and heart failure. This could, together with the collagen‐depositing signalling of fibroblasts under stretch overload, cause the established phenotype of systolic heart failure, where collagen production is high, but the crosslinking is diminished (Gunja‐Smith *et al*. 1996; Woodiwiss *et al*. 2001).

## From fibrosis to sympathetic activation

Diffuse fibrosis occurs as a consequence of pressure overload as described above, but it is also associated with normal ageing, contributing to progressive cardiac stiffening even in the absence of an obvious cardiovascular disease (Biernacka & Frangogiannis, 2011). Any stiffening might be expected to be associated with a loss of the ability both to contract and to relax due to a part of the work performed by myocytes being absorbed by the ECM (Brilla *et al*. 1996). However, in ageing humans, the contractile capabilities of a heart are often preserved even when the ability to relax is significantly impaired (Chen, 2009; Santulli & Iaccarino, 2016). This suggests the existence of a compensatory mechanism that allows the heart to increase its contractility so that cardiac output can be maintained.

One such compensatory mechanism could be the activity of the cardiac sympathetic nervous system, which is known to be pro‐inotropic, i.e. increasing contractile strength (Goldberg *et al*. 1960). Like fibrosis, increased activity of the sympathetic system is often considered pathological, as it is found in dilatation‐induced heart failure (Kyuma *et al*. 2004), and is a contributing factor in the incidence of ventricular fibrillation, especially after a myocardial infarction (Shen & Zipes, 2014).

Despite these observations, we believe that the studies below show that CSNS is a crucial feature allowing stiff hearts to maintain sufficient cardiac output and that in certain well‐defined circumstances, excessive inhibition of the sympathetic system may be dangerous, rather than beneficial.

An example of a medical condition that could be related to CSNS inhibition is sudden cardiac death (SCD) due to pulseless electrical activity (PEA), the incidence of which is increasing, while effective treatment strategies are lacking (Hallstrom *et al*. 2009). In PEA, the electrical signal is propagated normally, but no contraction occurs. A second medical condition is asystole, where both electrical conduction and contraction are absent. We note that both these conditions are likely to improve with bolstering cardiac sympathetic nervous activation (which is among the recommended therapies): in PEA, the inotropic effect of sympathetic stimulation is expected to improve the issue of insufficient contractility. In asystole, sympathetic stimulation is expected to improve conduction of electrical signals from pacemaking cells, as it makes myocytes more excitable overall via activation of fast sodium channels (Baba *et al*. 2004) and the L‐type calcium current (Herzig *et al*. 1993).

### Systems‐level evidence

An excellent human model of CSNS inhibition is heart transplantation, where the heart's natural innervation is disrupted (Vaseghi *et al*. 2009); here, denervation results in low levels of myocardial catecholamines, which persist for several years after transplantation (Regitz *et al*. 1990). In the normal population, the main cause of SCD is ventricular fibrillation (VF); however, in heart transplanted patients, VF is rare and the main causes of SCD are PEA and asystole (Vaseghi *et al*. 2009). This suggests that the denervation decreases the likelihood of a VF by negating the pro‐fibrillatory effect of CSNS, but the benefit of increased inotropy and facilitation of conduction is also lost, increasing the likelihood of PEA and asystole. It should be noted that transplanted hearts may be reinnervated, but this process is quite slow; as shown by Überfuhr *et al*. (2000), even after 4.6 years, 40% of transplant patients were classified as ‘not reinnervated’. The mean time between transplant and death in the study by Vaseghi was 3.5 years in non‐sudden deaths (and 1.3 years in sudden deaths), suggesting that these deaths could have happened before reinnervation took place and that the hearts can be indeed considered at least partially denervated.

A different model of CSNS inhibition is drug treatment, such as beta‐blockade. This is a much more complex model, however, since beta‐blockers are far wider‐acting than selective cardiac denervation. While beta‐blockade is very likely to decrease the intrinsic contractility of the heart by blocking pro‐inotropic catecholamine activity, it also decreases heart rate (thus improving diastolic filling) and usually decreases systemic blood pressure, decreasing overall cardiac workload (Frishman & Saunders, 2011). Therefore, beta‐blockade may not invariably introduce problems with contractility. It has been shown by Hall *et al*. (1995) that in cases of dilated cardiomyopathy (and thus systolic heart failure), beta‐blocker administration causes initial and transient decrease of cardiac output (proposed to be due to decreased inotropy), but the cardiac output then improves above the original value (proposed to be due to decreased cardiac workload). To our knowledge, there is unfortunately no such study for diastolic heart failure. However, the study by Youngquist *et al*. (2008) is perhaps indicative of the effect of beta‐blockers on patients with stiff hearts. The authors of this study have performed a study in aged patients (median 70 years, interquartile range 57–79 years) who are likely to have increased myocardial stiffness, demonstrating that within the observed group, beta‐blocker use was associated with a significant and large increase of likelihood of PEA over VF compared to patients without beta‐blockers. We note, however, that this study was an observational one and not randomized, i.e. the patients administered beta‐blockers might have had an *a priori* high likelihood of PEA. Nevertheless, as the authors state, this study suggests that widespread use of beta‐blockers might be the key factor in changing epidemiology of VF and PEA between the 1980s and now. While the incidence of VF as the initial rhythm in out‐of‐hospital cardiac arrest has decreased from 61–65% to 35–48%, the incidence of PEA grew to the current state of 22–30% (Saarinen *et al*. 2012).

Indirect evidence of the compensatory role of CSNS is also seen in the study by Grassi *et al*. (2009), which found significantly increased muscle sympathetic activity in hypertensive patients with diastolic dysfunction, compared both to hypertensive patients with normal diastolic function and to a control group of non‐hypertensive patients. At the same time, systolic function did not differ significantly between the three observed groups, as evaluated by ejection fraction and fractional shortening. If we assume that the diastolic dysfunction in the observed group was caused by increased cardiac stiffness, sustained systolic function is indicative of a compensatory mechanism that increases contraction strength: CSNS with its known pro‐inotropic effect, and being so clearly elevated in this study, seems a very likely candidate. One caveat is that this study measured striated muscle sympathetic activity, but it is known to be correlated to cardiac sympathetic activation (Lambert *et al*. 2011).

Animal studies also suggest the importance of the CSNS for maintaining sufficient contractility. One such example is the study by Albrecht *et al*. (1975), where sympathetic deactivation via pithing caused an almost 50% decrease in cardiac output, which could be only partially explained by decreased heart rate.

In summary, the existing literature suggests that in the presence of myocardial stiffness, increasing CSNS reduces the risk of PEA due to improved contractility, but at the same time, increases the risk of VF. This perspective may lead to a framework that can guide therapy: e.g. when a patient is treated for fibrillation with sympathoinhibitory drugs, the dose might be adjusted according to how stiff the patient's heart is.

## Myocardial infarction and pressure overload

In this section, we briefly comment on why the stretch‐sensing mechanisms described above are relevant also to the heart with healed myocardial infarction. After myocardial infarction, a stiff scar is eventually formed in place of the infarcted tissue (Czubryt, 2012). Being composed mainly of collagen, the scar is far less contractile than the surrounding myocardium (Fomovsky & Holmes, 2010); when it is not sufficiently firm, the infarcted tissue is prone to dilatation and/or rupture (Noppe *et al*. 2014).

When a segment of the myocardial wall becomes stiff, assuming constant internal volume, the remaining myocardium is stretched with greater force during diastole. In addition, in order to maintain cardiac output, the remaining healthy tissue must generate more force to compensate for the scar. This makes the presence of stiff scars in myocardium a sort of analogue of elevated systolic and diastolic pressure. Indeed, certain processes we described earlier as being linked to hypertension have been observed after myocardial infarction, i.e. increased angiotensin‐II (Ang‐II) secretion (Sutton & Sharpe, 2000) and subsequent fibrosis in non‐infarcted myocardium (Volders, 1993); the hyperinnervation of non‐infarcted myocardium has also been described (Zhou *et al*. 2004). It is possible that these changes are mediated largely by similar stretch‐sensing mechanisms as the ones discussed earlier in this text, with the same consequences of their imbalance. To our knowledge, there is no study researching the role of post‐infarction interstitial hyperinnervation in maintaining contractility, but the role of matrix metalloproteinases and their inhibition after MI has been studied in animal models. The results are largely consistent with the role of collagen dynamics in hypertension, where inhibition of degradation via MMP inhibition attenuates left ventricular dilatation (Rohde *et al*. 1999; Lindsey *et al*. 2002); this effect is not entirely due to the acute healing phase, but has a role in the late healing phase as well (Mukherjee *et al*. 2003).

## Summary and discussion

**Figure 2:**
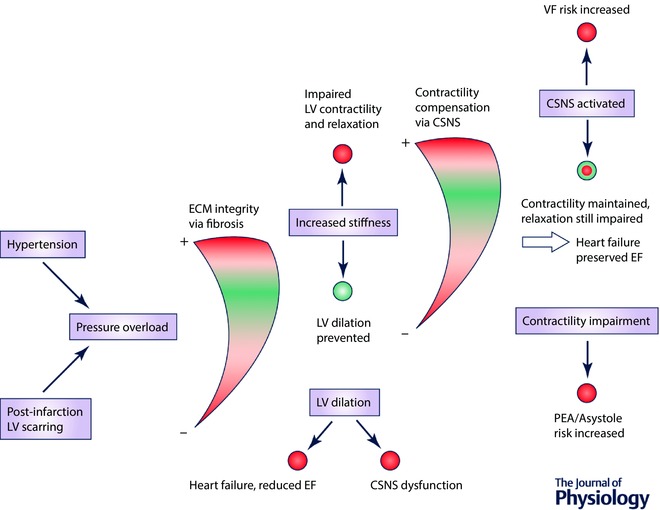
A model of disease development according to presence of remodelling steps Blue nodes represent concepts of heart disease, with some of their consequences given as coloured circles: red, detrimental; green, beneficial. The gauges represent a spectrum of their respective feature (how much ECM integrity is increased, or how much CSNS is activated), with the red representing detrimental extremes and green representing a ‘sweet spot’, where total risk of both extremes is minimized. EF, ejection fraction; LV, left ventricular.

Putting together the links between pressure overload and fibrosis and between fibrosis and CSNS, a simplified model of disease based on pressure overload can be synthesized, as shown in Fig. 2. This model provides additional insight to the observation that fibrosis is associated with occurrence of arrhythmias (Shiozaki *et al*. 2013). In addition to the role of increased conduction heterogeneity in a fibrotic environment, the model explains why fibrosis might be indirectly associated with an increase of cardiac sympathetic activity, promoting arrhythmias by a mechanism not directly linked to fibrotic remodelling.

We consider the model to be a useful tool in evaluating existing and developed therapies. For example, anti‐fibrotic therapy has been proposed to ameliorate fibrosis‐associated arrhythmias (Morita *et al*. 2014), which is indeed expected to decrease the occurrence of arrhythmias. However, our model suggests that if pressure overload is also present and is not targeted by the therapy, the outcome can be detrimental to patients’ health by a disproportionate increase of risk of myocardial dilatation. An example of a controversial therapy would be the potentiation of MMPs, which is expected to ameliorate fibrosis, but might diminish the structural integrity of myocardium. On the other hand, angiotensin converting enzyme (ACE) inhibitors or angiotensin receptor antagonists seem to be a safer way to treat fibrosis, as they not only act anti‐fibrotically (Ciulla *et al*. 2009), but also decrease blood pressure, which in turn reduces the stress on myocardial structure and thus the underlying pro‐fibrotic signalling. It is yet to be determined how much the anti‐fibrotic effect of ACE inhibitors is due to a direct effect and how much is due to prevention of stretch‐induced pro‐fibrotic remodelling via decreased blood pressure.

The model might also be relevant to the understanding of the two flavours of heart failure: systolic and diastolic. These are conditions that are frequently researched together (‘heart failure’) in clinical studies, but which are largely different diseases with different underlying causes and thus requiring an entirely different therapy (this topic is discussed in greater depth in Iwano & Little (2013)). For example, there are therapies shown to be efficient in systolic heart failure, but a successful therapy for diastolic heart failure is lacking (Udelson, 2011). According to the studies discussed in this article, both types of heart failure may be viewed as two sides of a single coin: ECM integrity.

An initial loss of ECM integrity, caused by a breakdown of collagen or reduced crosslinking, may result in dilatation and systolic heart failure. Myocardial stretch promotes CSNS activity, which initially improves contractility, but this can fail due to excessive CSNS overactivation and neurotransmitter depletion on β‐receptor downregulation (Port & Bristow, 2001), possibly via β‐arrestins (Bathgate‐Siryk *et al*. 2014). Interestingly, the benefit of beta‐blockers in systolic heart failure is partly due to restoration of beta‐receptor density, which then paradoxically potentiates CSNS (López‐Sendón *et al*. 2004). It remains an open question of how the loss of collagen integrity may be prevented in the first place: MMP inhibition has been proposed (King *et al*. 2003), but human mortality studies of MMP inhibitor therapy are lacking.

On the other hand, diastolic heart failure is caused by an increase in ECM integrity in our model, manifesting as increased myocardial stiffness (which might be either a physiological compensation of pressure overload or a pathological dysregulation). In this context, CSNS is elevated to maintain sufficient contractility, but there is a lack of evidence for activation and desensitization to the same degree as is seen in systolic heart failure, which could underlie the observation that beta‐blockers are not nearly as efficient in treating diastolic heart failure (Paulus & van Ballegoij, 2010). Our review suggests that the lack of overt CSNS dysregulation in diastolic heart failure could be due to myocardial stiffness limiting myocardial stretch, preventing excessive pro‐CSNS signalling based on stretch sensing. Several studies cited in this review indirectly suggest that when anti‐inotropic drugs are administered to patients with diastolic heart failure, the degree of cardiac stiffness should be taken into account. Among the recommended treatment of diastolic heart failure are symptom relief and an aggressive treatment of hypertension (Rose‐Jones *et al*. 2014), which is again an interesting link to the model synthesized in this review, where diastolic heart failure is predicted to happen as a consequence of hypertension. From the point of future therapy, a possible research direction is a cardiac‐specific activation of certain MMPs with the motivation of reduction in cardiac stiffness. However, this would require a careful balancing of breakup of collagen and its crosslinks while maintaining the necessary structural integrity of ECM to prevent dilatation and/or rupture.

Hypertension and heart failure are complex systemic diseases with a vast range of possible interactions. In the current review we focus on the relationship between ECM integrity and CSNS, but many other factors play significant roles. For example, sustained β‐adrenergic stimulation is known to induce hypertrophy via several mechanisms (e.g. Sorriento *et al*. 2010) and activates G‐protein receptor kinases that have diverse effects (e.g. induction of insulin resistance, Santulli *et al*. 2013), which limits the ability of CSNS to compensate for the effects of heart failure. In addition, cell coupling modulators (Egan Benova *et al*. 2016) and immune regulators (González *et al*. 2016) have recently been identified as promising therapeutic targets that leverage different pathways from those discussed here. More research is needed to determine the relative importance of these interactions in disease progression. Nevertheless, we hope that this review provides insights and may eventually lead to new strategies for disease management and treatment.

## Additional information

### Competing interests

None declared.

### Author contributions

Both authors have approved the final version of the manuscript and agree to be accountable for all aspects of the work. All persons designated as authors qualify for authorship, and all those who qualify for authorship are listed.
